# Cubic Air Space for Institutional Infants

**Published:** 1915-10-30

**Authors:** 


### CUBIC AIR SPACE FOR INSTITUTIONAL INFANTS.

Some conclusions which are important to architects and others concerned with hospital planning and administration have been arrived at in the United States by Dr. Southworth, as the result of a canvass among a large number of the leading experts in child study and children's diseases.*

Infants, he says, should have as much cubic air space allowed them as adults: and this is especially true of bottle-fed infants, because they are more susceptible to infection, suffer in their nutrition in confinement, and "notoriously do badly in all save the best-equipped institutions." We do not believe that this last sweeping stricture is justifiable in this country, and we rather doubt whether it is not too scathing also with regard to American institutions. On account of their handicaps, bottle-fed infants are liable to require more space than nursing infants: thus far, at any rate, there will be agreement with Dr. Southworth, who finds that the bottle-fed want as much space as is required by sick infants, whether in medical or surgical wards. He puts the minimum requirement at 1,000 cubic feet per child: some institutions provide only 600 cubic feet, an allowance which he deems insufficient. Furthermore, even when 1,000 cubic feet are provided, free ventilation is highly important; and should the space allowed be less, then ventilation becomes more than ever imperative.

In emphasising his declaration that bottle-fed infants want as much space as if they were ill, Dr. Southworth says that when retained for any considerable length of time in hospitals or institutions, bottle-fed infants cannot properly be classed as well because a considerable proportion of them suffer both in their digestion and nutrition, as well as from acute intercurrent infections. Here again the author seems to be exaggerating matters: no one doubts that breast-fed children thrive better than bottle-fed, but to talk as if the bottle-fed were one and all peaked and ill is simply nonsense. It would be a great pity if these manifest over-statements should cause to be overlooked some of his other conclusions, which can be fully commended. Thus he points out that the mere presence of windows does not of itself constitute ventilation, unless they are properly used. He also lays stress upon the value of roof gardens, porches, and sun-parlours in the design of institutions for infants.

* *Archives of Pediatrics*, September 1915, p. 641.

